# To investigate the prevention of OM-85 on bronchiectasis exacerbations (iPROBE) in Chinese patients: study protocol for a randomized controlled trial

**DOI:** 10.1186/1745-6215-15-150

**Published:** 2014-04-29

**Authors:** Jinming Gao, Xiang Gao, Lingfei Kong

**Affiliations:** 1 Department of Respiratory Diseases, Peking Union Medical College Hospital, Chinese Academy of Medical Sciences & Peking Union Medical College, #1 Shuaifuyuan, Dongcheng District, Beijing 100730, People’s Republic of China; 2 Channing Laboratory, Department of Medicine, Brigham and Women’s Hospital, Harvard Medical School, 181 Longwood Ave, Boston, MA 02115, USA; 3 Department of Nutrition, Harvard University School of Public Health, 677 Huntington Ave, Boston, MA 02115, USA; 4 Department of Respiratory Diseases, The First Hospital of China Medical University, #155, Nanjing Street North, Shenyang 110001, Liaoning Province, People’s Republic of China

**Keywords:** OM-85, Bronchiectasis, Placebo, Pulmonary exacerbation

## Abstract

**Background:**

Non-cystic fibrosis bronchiectasis is characterized by the irreversible dilatation of the medium-sized bronchi as a result of airway injury from recurrent or chronic inflammation and lower respiratory tract infections. Bronchiectasis airways are commonly colonized with bacterial species. Infections of the airways play important role in bronchiectasis exacerbations. The non-specific prevention of recurrent airway infections by immunostimulating agents has gained growing interest. OM-85, consisting of extracts of eight kinds of bacteria important in respiratory infections, could support the respiratory tract resistance to the pathogens. OM-85 has been shown to be a benefit by decreasing the risk of acute exacerbation of chronic obstructive pulmonary disease (COPD) in several perspective clinical trials. Exacerbation of bronchiectasis substantially contributes to a more rapid decline in lung function, reduced quality of life, and healthcare costs. In this context, we plan to conduct a clinical trial to investigate the PReventive effect of OM-85 on Bronchiectasis Exacerbation in Chinese patients (iPROBE).

**Methods/Design:**

This study is designed as a prospective, randomized, double blind, placebo-controlled multicenter trial. A total of 244 patients with bronchiectasis, who have had at least one exacerbation of bronchiectasis in the previous year, will be included. The subjects will randomly receive two courses of 7 mg of OM-85 or a matching placebo. The treatment dose of OM-85 will be one daily capsule taken orally for 10 days each month for 3 consecutive months at the beginning of the study, followed by 3 months of no drug. This schedule will repeat until the patient has been seen for one year.

**Discussion:**

We will investigate whether long-term treatment with an oral immunostimulant (OM-85) could decrease exacerbations of bronchiectasis over a one-year period. We will also assess other relevant outcomes, including the rate of event-based exacerbation, lung function parameters, and total scores judged by the St George’s respiratory questionnaire, Leicester cough questionnaire, and inflammatory index. We hope that this study will provide new information on the preventive effects of OM-85 on bronchiectasis exacerbations and will address a knowledge gap for this understudied disease.

**Trial registration:**

This study is registered at http://www.clinicaltrials.gov (identifier NCT01968421) on 19 October 2013.

## Background

Bronchiectasis is a common, however, under-recognized chronic respiratory disease [1-3]. The prevalence of bronchiectasis in adult populations worldwide is unknown [3,4]. In the United States, an estimated prevalence of bronchiectasis in adults was 52 in every 100,000 people, and increased by 8.7% per year between 2000 and 2007 [5]. Non-cystic fibrosis bronchiectasis is pathologically characterized by an irreversible dilatation of the medium-sized bronchi as a result of airway injury due to recurrent or chronic inflammation and infection. Clinical features of bronchiectasis include chronic production of sputum (often mucopurulent or purulent in nature), persistent bacterial colonization, recurrent lower respiratory tract infections, and hemoptysis, with breathlessness characterized by mild to moderate airflow obstruction, lethargy, and reduced health status [2].

Bronchiectatic airways are often colonized with bacterial species such as *Haemophilus influenzae, Streptococcus pneumoniae* and *Pseudomonas aeruginosa*[2,6]. Thus, it is believed that infections of the airways frequently play an important role in bronchiectasis exacerbations [7]. Macrolide antibiotics with anti-inflammatory and immunomodulatory properties are increasingly prescribed for subjects with this disease [8]. Recently, Wong *et al.* demonstrated that azithromycin three times a week for six months significantly decreases the incidence of bronchiectasis exacerbations [9]. However, widespread use of long-acting macrolides, particularly azithromycin, risks significantly increasing population antimicrobial resistance as well as potential adverse drug effects [10].

Abnormalities in innate and adaptive immunity may cause a predisposition to bronchiectasis. Progressive lung damage in bronchiectasis results from a ‘vicious cycle’ of recurrent bacterial infection and a deregulated inflammatory response. This indicates the failure of the host defenses that have evolved to maintain the sterility of the respiratory tract [7,11]. The non-specific prevention of recurrent airway infections by immunostimulating agents has gained growing scientific interest over the past 40 years and is increasingly applied in different clinical situations [12].

OM-85 has been known to support the respiratory tract resistance to pathogens. It consists of extracts of eight kinds of bacteria thought to be important in respiratory infections. OM-85 does not appear to be a bacterial vaccine but is thought to function by activating pulmonary macrophages, increasing the ratio of CD4 to CD8 lymphocytes, and changing the level of a variety of cytokines in the lung. The changes are thought to be indicative of an increased level of host defense against a wide variety of pathogens including both viruses and bacteria [12]. Thus, the protective effect of OM-85 implicates infection as an important player in the pathogenesis of exacerbations but does not indicate a specific class of infectious agent. As such, the potential benefit of OM-85 has been evaluated in a variety of chronic respiratory diseases. The best studied condition is chronic obstructive pulmonary disease (COPD), in which there have been several randomized controlled trials showing evidence of benefit by significantly reducing the incidence of exacerbations of COPD of all severities [13,14]. Bronchiectasis exacerbations contribute substantially to rapid decline in lung function, reduced quality of life, and the medical costs. In patients with bronchiectasis, exacerbations occur with a frequency rate of 2.6 exacerbations annually [15].

The purpose of this multicenter, randomized, controlled trial is to investigate the PReventive effect of OM-85 on Bronchiectasis Exacerbations in adult Chinese patients (iPROBE). The iPROBE project is generally based on the concept that the majority of patients with bronchiectasis often suffer from exacerbations.

### Aims of the study

**Figure 1:**
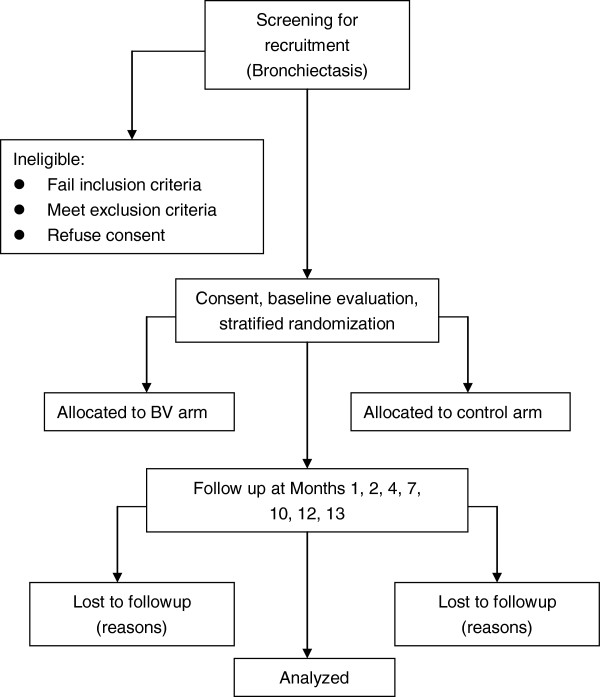
**Flow chart of the patients through the clinical trial.** BV: broncho-vaxom (OM-85).

This study is structured as a multicenter, randomized, double blind, placebo-controlled trial (RCT) to study the effect of treatment with OM-85 in preventing acute exacerbation in patients with bronchiectasis (Figure 1).

The primary goals of this study include two domains: (1) a reduction in the numbers of acute exacerbation; and (2) percentage of patients free of event-based exacerbation after one year.

The secondary efficacy variables are St George’s Respiratory Questionnaire (SGRQ); Leicester Cough Questionnaire (LCQ); use of antibiotics and rapid-acting bronchodilator; lung function parameters: forced expiratory volume in first second (FEV1) predicted, forced vital capacity (FVC), FEV1/FVC, immunoglobulin (Ig), total immunoglobulin E (TIgE), rheumatic factor (RF); C-reactive protein (CRP) in serum; and sputum bacteria clearance.

## Methods/Design

### Overall design and patient selection

**Figure 2:**
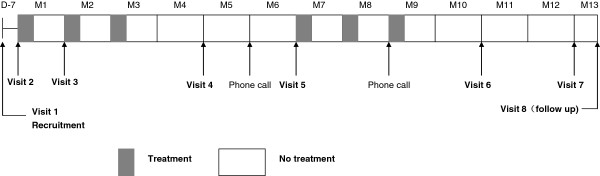
Treatment periods and visits.

This RCT study will be performed at 10 major medical centers in Beijing, Shenyang, Tianjin, Zhejiang, and Shanghai, China. We plan to recruit 244 patients with bronchiectasis diagnosed using high-resolution computed tomography (HRCT). The patients should have had at least one exacerbation requiring antibiotics in the previous year prior to recruitment. Participants will be invited to visit the clinical centers on six occasions and follow up will be conducted by telephone on two occasions during the year (Figure 2). The investigations to be performed are shown in Table 1.

**Table 1:** Overview of clinic visits and measurements

	**Day 1**	**Month 1**	**Month 3**	**Month 5**	**Month 7**	**Month 9**	**Month 12**
Informed consent	×						
Demographic data	×						
Blood works	×	×		×			×
Leicester cough score	×	×	×	×	×	×	×
SGRQ score	×	×	×	×	×	×	×
Lung function test	×			×			×
Sputum microbiology	×			×			×

SGRQ: St George’s respiratory questionnaire.

An exacerbation will be defined as the patient reporting four or more of the following symptoms: change in sputum production, increased dyspnea, increased cough, fever over 38°C, increased wheezing, decreased exercise tolerance, fatigue, malaise, lethargy, reduced pulmonary function, changes in chest sounds or radiographic changes consistent with a new infectious process [3].

The study will occur over a three-year period. Recruitment of cases and controls to the study will be performed over a two-year period (May 2013 to May 2015). The treatment period will last for one year. The end of the study is defined by the last visit of the last participant (Figure 2).

### Randomization

All eligible subjects will be randomized using block randomization sequences generated by computer. The subjects will be assigned at a 1:1 ratio with a permuted block size and sequential assignment, stratified by the center. Treatment allocation numbers will be entered into individually sealed envelopes containing a number that is concealed to the treatment allocation. The allocation list will be stored in a safe place in each study site and accessed by a non-investigator independently. In the event of a medical emergency the individual’s randomization code and group allocation could be identified.

### Intervention

The subjects will randomly receive two courses of 7 mg of OM-85 or matching placebo. The treatment dose of OM-85 of will be one daily capsule taken orally for 10 days each month for 3 consecutive months at the beginning of the study, followed by 3 months of no drug. This schedule will repeat, and then be followed until the patient has been seen for one year. During the whole study period, subjects will be followed at the outpatient clinics 1, 2, 4, 7, 10, and 12 months after initiating the study (Figure 2). During the visits, Table 1 shows the tests that will be performed.

### Eligibility criteria

Participants were eligible for inclusion in the study when they are 18 years or older diagnosed with bronchiectasis by HRCT of the chest within three years of study inclusion, and having had at least one acute exacerbation in the previous year when recruiting.

Exclusion criteria include history of cystic fibrosis; hypogammaglobulinemia; allergic bronchopulmonary aspergillosis; active tuberculosis; subject has been assigned to treatment in a study of the medication under investigation in this study in the previous six months; subject has a history of chronic alcohol or drug abuse within the last six months judged by the investigators; hypersensitivity to any components of OM85; subject has received an immunostimulant in last three months; cancer; stroke; severe cardiovascular disease; hepatic and/or kidney impairment, and immuno-related diseases**.**

Subjects are free to withdraw from the study at any time, without prejudice to their continuing care.

Investigators should attempt to obtain information on subjects in the case of withdrawal or discontinuation. For subjects considered as lost to followup the investigator should make an effort (at least one phone call and one written message to the subject), and document their effort (date and summary of the phone call and a copy of the written message in the source documents) to complete the final evaluation. All results of these evaluations and observations, together with a narrative description of the reason(s) for removing the subject, must be recorded in the source documents. The case report form must document the primary reason for withdrawal or discontinuation. Investigators should contact the Medical Monitor whenever possible to discuss the withdrawal of a subject in advance.

Written informed consent approved by an Institutional Review Board (IRB) or Independent Ethics Committee (IEC) is signed and dated by the subject.

### Sample size

To detect a true difference in the primary outcome measures of the number of bronchiectasis exacerbations and the time to first exacerbation, a total of 244 subjects (122 per arm) will be required. This calculation is based on the 80% probability to detect a 33% reduction in the numbers of bronchiectasis exacerbation and a significantly greater median time to the first exacerbation by OM-85 treatment. The incidences of bronchiectasis exacerbation were reported to be between 1.5 and 6.5 times per patient per year [3,16], and median time to first time to exacerbation to be 85 days in a clinical trial lasting over a one-year period [9].

### Statistics

There are three analysis populations in the study: full analysis set (FAS), per protocol set (PPS) and safety set. The primary endpoint will be analyzed on FAS, which is the primary population, and will be supported by PPS. The secondary endpoint and baseline information will be analyzed on FAS. Safety analyses will be performed on the safety set.

The primary efficacy variable is the numbers of acute exacerbation during the treatment. It will be analyzed on FAS population and supported by PPS population. Statistical description and statistical inference will be conducted. Mean, standard deviation, median, maximum and minimum will be presented as a statistical description for each study group.

Statistical inference consists of a parameter estimation and hypothesis test. For categorical variables, frequency and proportion will be summarized by group. Comparison between two groups will be performed using Student-t test or Wilcoxon test depending on data distribution. A two-tailed 95% confidence interval of the difference between groups will also be presented.

The categorical data will be analyzed by the Pearson chi-square test or Fisher exact test. The change before and after treatment within each group will be described and tested by the paired-t test or signed-ranks test.

The interval to the first time of acute exacerbation will be analyzed as survival data. Kaplan-Meier curves of two groups will be presented and thelog-rank test will be used to test the difference between two groups.

All analyses will follow the intention-to-treat principle. We will also employ the following methods to minimize the potential effects of missing data or dropout. First, when the study treatment is discontinued, we will try to obtain consent from the participants to collect data on treatments and outcomes. W we will use an estimating-equation method to impute missing data [17] in our primary analysis; and we will conduct a sensitivity analysis by using simple imputation methods.

### Safety analysis

Analysis on safety will be done using the safety set. All adverse events will be coded using the last version of Medical Dictionary for Regulatory Activities. Total frequency and incidence of adverse event (AE) and severe AE (SAE) will be described. The frequency of AE in each group will be tabulated by system organ class and the preferred term. The severity and relationship with the study will be summarized by each group. AE-related (definitely related, probably related, and possibly related) will be described and listed separately by the two groups. AE leading to withdrawal from the study will be described and summarized separately by the two groups.

### Ethical approval

The study has been approved by the Central Ethics Committee of Peking Union Medical College Hospital. The detailed study will be explained to the patient prior to enrolment in the study by the investigator. The explanation will include the type, the purpose, the tests to be performed, and any potential hazards. A written informed consent will be obtained from each participant. The patient can withdraw from the study at any time, without any repercussion for the ongoing care.

The study will be conducted according to the International Conference for Harmonization (ICH) principles of Good Clinical Practice (GCP) and the Declaration of Tokyo (2004). The investigator will conduct all aspects of this study in accordance with all national and regional laws of the pertinent regulatory authorities.

## Discussion

With the widespread availability of chest HRCT, bronchiectasis is becoming increasingly recognized. It has been concluded that bronchiectasis remains a common and important cause of respiratory disease [4]. There is an overlap between COPD and bronchiectasis, with a reported incidence of bronchiectasis in COPD being between 29% and 50% [18]. There are also reports of a high prevalence in relatively isolated populations with poor access to health care and high rates of respiratory tract infection during childhood [19]. However, treatment of bronchiectasis is unsatisfactory with only very small trials being conducted, and there are currently no disease-modifying drugs. Immunomodulation agents such as low-dose macrolides have also been shown to have some efficacy, although more data are needed to advocate their long-term usage. Some studies have shown that chronic azithromycin treatment increases the development of macrolide-resistant organisms such as *S. pneumoniae*[10]. However, azithromycin could also predispose patients with cystic fibrosis to the development of non-tuberculous mycobacterial infection [9]. In addition, there is new evidence that the combination of azithromycin and tobramycin, through the alteration of intracellular antibacterial activity, may lead to even worse bacteriological outcomes. However, recent evidence has shown that azithromycin may antagonize inhaled tobramycin when targeting *P. aeruginosa* in cystic fibrosis [20]. Finally, an optimum duration of treatment with azithromycin is unclear.

A possible mechanism for bronchiectasis is a significant infection in early childhood which causes structural damage to the developing lung, allowing perpetual bacterial infection. Over time, persistent infection may then result in chronic inflammation, resulting in tissue damage and impaired mucociliary motility. This in turn leads to more infection, with a cycle of progressive inflammation, causing lung damage. Based on the ‘vicious cycle’ model of the pathophysiology of bronchiectasis, persistent infection and a defect in host defense are two factors for the development of this condition [7,11].

Some observations have shown that airway inflammation in bronchiectasis is due to a deregulated cytokine network independent of bacterial airway colonization [7]. The iPROBE study is taking an approach that is distinct from other studies using antibiotics (oral, nebulized) to reduce the bacterial load and then decrease lower airway inflammation [9,19,21]. OM-85 would be used to improve host defense and reduce airway inflammation, allowing airway healing and thus modifying the long-term course of disease. The rationale of the current study for choosing OM-85 includes its immunomodulatory property and potential activity based on common respiratory tract bacterial isolates. OM-85 is not a new drug, having apparently been available in Europe over 40 years with a very good safety profile. OM-85 is relatively easy to administer, so it is likely that patient compliance would be as good as it is with mucolytic therapy.

The variables (primary and secondary) chosen in this study reflect the nature of bronchiectasis, and have been widely adopted by the majority of clinical trials on bronchiectasis [22,23].

In summary, this doubleblind RCT examining the prevention of OM-85 against exacerbation of bronchiectasis addresses a gap in the knowledge and treatment for this neglected disease. If the intervention is efficacious, it would provide long-term benefits for patients with bronchiectasis and improve the prognosis of this condition.

## Trial status

The trial is scheduled to begin recruiting participants from May 2013, and expected to complete the enrollment by May 2015.

## Competing interests

The authors declare that they have no competing interests.

## Authors’ contributions

JG: conception and design, data collection and analysis, manuscript writing and final approval of the manuscript. XG: data collection and analysis, critical revision and final approval of the manuscript. FK: design, initiation, critical revision and final approval of the manuscript. All authors read and approved the final manuscript.
